# An Analytical Approach for Naturalistic Cooperative and Competitive EEG-Hyperscanning Data: A Proof-of-Concept Study

**DOI:** 10.3390/s24102995

**Published:** 2024-05-09

**Authors:** Gabriella Tamburro, Ricardo Bruña, Patrique Fiedler, Antonio De Fano, Khadijeh Raeisi, Mohammad Khazaei, Filippo Zappasodi, Silvia Comani

**Affiliations:** 1 Behavioral Imaging and Neural Dynamics Center, G. d’Annunzio University of Chieti-Pescara, 66100 Chieti, Italy; antonio.defano@unich.it (A.D.F.); filippo.zappasodi@unich.it (F.Z.); comani@unich.it (S.C.); 2 Department of Neuroscience, Imaging and Clinical Sciences, G. d’Annunzio University of Chieti–Pescara, 66100 Chieti, Italy; khadijeh.raeisi@unich.it (K.R.); mohammad.khazaei@unich.it (M.K.); 3 Center for Cognitive and Computational Neuroscience (C3N), Universidad Complutense de Madrid, 28040 Madrid, Spain; ricardo.bruna@ucm.es; 4 Department of Radiology, Rehabilitation and Physiotherapy, School of Medicine, Universidad Complutense de Madrid, IdISSC, 28040 Madrid, Spain; 5 Institute of Biomedical Engineering and Informatics, Technische Universität Ilmenau, 98693 Ilmenau, Germany; 6 Institute for Advanced Biomedical Technologies, University “Gabriele d’Annunzio” of Chieti–Pescara, 66100 Chieti, Italy

**Keywords:** hyperbrain analysis, electroencephalography, cooperation, competition, intra-brain coupling, inter-brain coupling, joint action, table tennis

## Abstract

Investigating the neural mechanisms underlying both cooperative and competitive joint actions may have a wide impact in many social contexts of human daily life. An effective pipeline of analysis for hyperscanning data recorded in a naturalistic context with a cooperative and competitive motor task has been missing. We propose an analytical pipeline for this type of joint action data, which was validated on electroencephalographic (EEG) signals recorded in a proof-of-concept study on two dyads playing cooperative and competitive table tennis. Functional connectivity maps were reconstructed using the corrected imaginary part of the phase locking value (ciPLV), an algorithm suitable in case of EEG signals recorded during turn-based competitive joint actions. Hyperbrain, within-, and between-brain functional connectivity maps were calculated in three frequency bands (i.e., theta, alpha, and beta) relevant during complex motor task execution and were characterized with graph theoretical measures and a clustering approach. The results of the proof-of-concept study are in line with recent findings on the main features of the functional networks sustaining cooperation and competition, hence demonstrating that the proposed pipeline is promising tool for the analysis of joint action EEG data recorded during cooperation and competition using a turn-based motor task.

## 1. Introduction

In everyday life, people often have to coordinate their actions with those of others in time and space to achieve a shared and public goal that cannot be achieved by acting individually [1]. This form of interpersonal interaction, referred to as joint action [2], is characterized by a strict bidirectional interdependence between interacting individuals’ actions and goals [3,4]. However, within a joint action, people are also driven by individual goals that can be either mutual and complementary (i.e., cooperation) or mutually exclusive (i.e., competition) [5,6]. Investigating the neural mechanisms underpinning cooperative and competitive joint actions may have a wide impact in many social contexts, such as education, business, industry, and sports, from simple actions to more complex relationships with mates, colleagues, and competitors, within and between genders [7,8].

The initial approach to the neuroscientific study of joint action was based on the recording and analysis of individual brain activations. However, this stand-alone approach was not suitable to capture the neurophysiological mechanisms underpinning the inherent mutual interdependence characterizing joint action [9,10,11]. Therefore, neuroscientists introduced the hyperscanning approach to the investigation of inter-brain functional interactions [12,13,14,15]. Nowadays, EEG is considered the most suitable non-invasive neuroimaging technique to study the rapid changes occurring in the brains of people involved in a joint action [12,15,16,17,18,19] because of the high temporal resolution of EEG recordings in the millisecond timescale [20,21]. Thanks to the availability of wireless, mobile, and lightweight EEG devices, the recording of brain activity in ecological and naturalistic environments during the performance of unconstrained tasks involving gross full-body movements is now possible [22,23]. However, despite the availability of this advanced EEG technology, hyperscanning studies have, to date, mainly employed very constrained paradigms simulating real-life situations, because they permit better control of the experiments and ensure high-quality EEG, as in cases of music performance [24,25,26], social games [27,28,29], flight simulations involving pilot and co-pilot teamwork [28,30,31], innovative employees’ evaluation in couples of manager–collaborator [32], and jugglers’ teamwork [22,33].

Importantly, most hyperscanning research has focused on cooperative joint action, because it encompasses diverse social contexts and the studies can rely on established analytical methods. Also, given the difficulty in identifying tasks that can be both cooperative and competitive, the neural mechanisms sustaining these two types of joint action have mostly been investigated through comparing the results obtained using different joint tasks, introducing a methodological bias. Only a few studies have investigated the neural mechanisms underpinning both cooperation and competition by employing the same task, although not within an ecological study paradigm [34,35,36,37]. Liu and colleagues [34] tried to concurrently investigate the neural mechanisms of cooperation and competition by employing the same quasi-ecological task for both conditions, i.e., tennis played through a video game. Despite the successful implementation of both conditions, this paradigm strongly differed from a real tennis match in terms of the nature of gameplay, level of physical activity, sensory feedback and perception, and the mode of social interaction. Additionally, the design of the cooperation condition, where both players collaborated against computer-simulated opponents, hindered a clear distinction between cooperative and competitive dynamics. Hence, the results obtained by Liu and colleagues [34] are not representative of real-world tennis.

According to the recent recommendations of social neuroscience [38,39,40], joint action should be investigated in naturalistic settings involving free full-body movements of the interacting individuals. However, there is, to date, no consensus or guidelines on the ideal analytical method(s) to identify the specific features of cooperative and competitive global brain dynamics and brain-to-brain coupling. The analysis of EEG–hyperbrain data usually relies on traditional neuroscientific methods such as functional connectivity and synchronization metrics (e.g., synchronization likelihood, phase synchronization index (PSI), phase locking value or phase lag index [19,41], and topological organization measures to asses features of the functional networks (e.g., graph theoretical measures of integration/segregation of the functional networks [22,33]). These approaches are appropriate to analyze the functional dynamics within one individual brain or between brains during the performance of cooperative, simultaneous, and continuous joint action tasks [24,31,42,43], but may be unsuitable to detect the functional dynamics between the brains of individuals involved in competitive and/or turn-based tasks. As elucidated in two recent studies [34,36], phase synchrony-based methods may face challenges in scenarios characterized by substantial variability in the frequency of the actions performed by the interacting individuals, as occurs in turn-based joint actions, especially when characterized by competitive elements. For instance, in tennis games, action frequency is highly variable and the players coordinate their movements while strategically aiming to disrupt this coordination for a competitive advantage. The unpredictability and variability of these competitive and turn-based actions pose challenges for the analysis strategies.

We recently proposed a novel study protocol including free full-body movements, to meet the recommendations of social neuroscience. We selected table tennis as the joint action task, as it permits implementation of a naturalistic experimental setting and can be played in both cooperative and competitive mode [44]. Notably, table tennis is a turn-based dyadic task that extends the variability of action frequency to both competition and cooperation. Therefore, analytical methods extensively used in EEG-hyperscanning studies may not be adequate to detect the characteristic features of the inter-brain neural dynamics and to differentiate between interaction conditions.

In the present study, we propose an analytical pipeline suited for EEG-hyperscanning data acquired during a naturalistic protocol employing a turn-based full-body motor task performed in both cooperative and competitive mode. The pipeline was designed to quantify the global intra- and inter-brain dynamics sustaining these two modes of the same turn-based joint action, hence contributing to filling a gap in the investigation of the neural mechanisms underpinning turn-based joint actions during real-world unconstrained cooperation and competition. To test the effectiveness of our pipeline, we performed a proof-of-concept EEG-hyperscanning study on two dyads according to our recently published table tennis study protocol [44]. Herein, we present the proposed analytical pipeline and the case study and compare our initial results with the literature. In future, following this successful proof of concept, we aim to use the positively validated study setup, paradigm, and analytical pipeline for an extended dataset including EEG-hyperscanning data from more dyads.

## 2. Materials and Methods

### 2.1. Participants

Four non-professional right-handed table tennis players (2 females aged 19 and 21, and 2 males aged 18 and 31) forming two same-gender dyads (one female and one male dyad) were recruited for our proof-of-concept study. They regularly practiced physical activities, did not report any neurological, psychological, or dermatological pathologies, and were not under pharmacological treatment. Written informed consent was obtained from each participant after explanation of the study. The study complied with the ethical standards outlined in the Declaration of Helsinki and was approved by the local Ethics Committee (Ethics Committee of Chieti and Pescara (Italy), meeting minutes N.06 of 11/03/2021).

### 2.2. Experimental Paradigm

EEG-hyperscanning acquisitions were performed according to our recently published table tennis protocol, that permits implementing both cooperation and competition within the same experimental framework [44]. Within this framework, the members of a dyad were asked to either exchange the ball as they do before a match (cooperation condition) or play table tennis like in a conventional match (competition condition). With this approach, both cooperative and competitive joint actions are implemented in an ecological environment, using the same motor task, with the interacting individuals making the same movements and having similar skills. This situation makes table tennis an ideal means for investigating and comparing the brain dynamics sustaining cooperation and competition.

Table tennis sessions took place in a laboratory room (9 × 6 m) with a professional table tennis table (Cornilleau Table Tennis 500 indoor/outdoor, 274 × 152.5 × 76 cm; 69 kg, Cornilleau SAS, Bonneuil-les-Eaux, France) positioned at the center of a rectangular area of about 54 m^2^. The two dyads were engaged in a total of 15 table tennis sessions of 30 s duration each (5 cooperative table tennis sessions and 10 competitive table tennis sessions), in randomized order. During the cooperative sessions, participants were instructed to keep the ball within the playing area for as long as possible. During the competitive sessions, participants had to play as in a real table tennis match. If the ball fell during a session, participants were instructed to catch another ball and restart the exchange until the end of the session. Participants were allowed to take short breaks between sessions. The higher number of competitive sessions (double than for the cooperative condition) is justified by the relatively lower number of rallies with a duration sufficient for the subsequent analysis, as described in detail in our study protocol [44].

### 2.3. Simultaneous Dyadic EEG Recordings

For the dyadic EEG recordings, we used our recently proposed and validated multimodal acquisition setup [44], allowing the simultaneous recording of two EEGs and video streams. EEG was recorded from each volunteer using a gel-based 61-channel setup with extended 10–20 layout and unipolar CPz reference (CA-205 waveguard original, ANT Neuro b.v., Hengelo, The Netherlands) in combination with a medically certified mobile EEG amplifier (EE-225 eego sports, ANT Neuro b.v.). EEG data were acquired at a sampling rate of 1024 samples/s. The anti-alias filter of the amplifier had a cut-off at 1/3 of the sampling frequency. The device integrates active shielding on all channels to reduce susceptibility to cable movement. Details on the EEG dataset are provided in Table 1.

**Figure 1:**
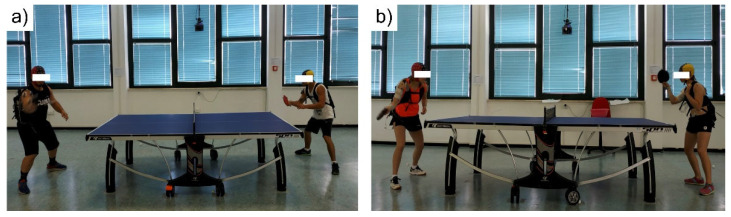
Setup of simultaneous dyadic EEG recordings: (**a**) male dyad; (**b**) female dyad.

Additionally, two video cameras, synchronized with the EEG mobile systems, captured digital videos at 25 fps. The videos were used for the definition of the rally segments. During the recordings, system control, remote data monitoring, data synchronization, and event distribution were ensured by our custom centralized setup as described in Tamburro et al., 2023 [44]. Figure 1 shows the experimental setting for the two dyads recruited for the proof-of-concept study. Note that each volunteer was wearing a backpack to house the EEG amplifier.

### 2.4. EEG Data Analysis

**Figure 2:**
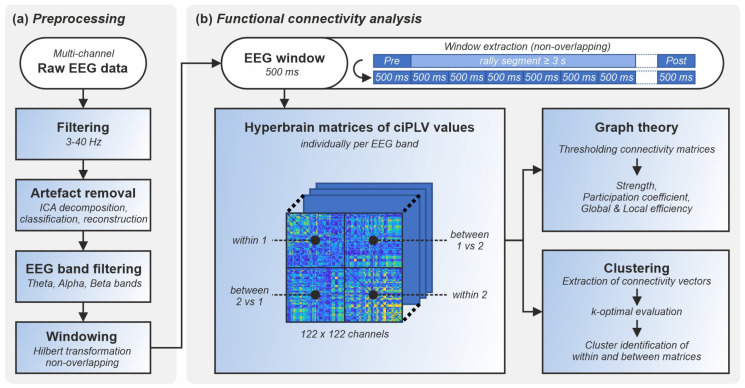
Visual representation of the proposed EEG analysis pipeline including two phases of (**a**) preprocessing and (**b**) functional connectivity analysis via calculation of hyperbrain matrices of the ciPLV values individually per band and window with subsequent graph theory and clustering analysis. The extraction of the analysis windows of 500 ms length each out of the overall rally segment ≥ 3 s length is shown on the upper right side.

To highlight differences in the neural dynamics and brain-to-brain coupling of real-world cooperation and competition, we employed a combination of methodological approaches. The analytical procedure consisted of 2 main steps: (1) EEG data preprocessing, including artifact removal, filtering in 3 frequency bands of interest, and windowing of the EEG signals; (2) estimation of functional connectivity maps, graph theoretical metrics, and functional clusters. Figure 2 shows the pipeline of analysis.

All analyses were performed using MATLAB (release R2018b; MathWorks, Natick, MA, United States) and the EEGLAB toolbox (release 14.1.1b; [45]). Computation of graph theoretical measures relied on formulas implemented in the Brain Connectivity Toolbox (https://sites.google.com/site/bctnet/, accessed on 3 January 2024, Sydney, Australia).

#### 2.4.1. EEG Data Preprocessing

EEG signals were band-pass filtered between 3 and 40 Hz using a zero-phase Hamming-windowed sinc FIR filter, implemented with the firfilt EEGLAB plugin [46]. By doing so, we excluded most movement related artefacts from the recordings and kept the information on brain activations in the frequency bands of interest, i.e., theta, alpha, and beta. In table tennis, these frequency bands are usually investigated for different reasons: the theta waves are associated with drowsiness, can be sometimes produced by hyperventilation, and can be facilitated by particular emotional states or mental processes of problem-solving; alpha waves are characteristic of mental relaxation, which can occur in expert athletes; beta waves are typical of active thinking and concentration, and are related to mental and cognitive processes of various types, anxiety, and state of alert.

Synchronous EEG recording sessions for the members of each dyad were determined using the start and stop events in the acquisitions. Within each recording session, the EEG segments corresponding to table tennis rallies (i.e., the actual ball exchanges) were identified using the videos, permitting us to visually define the instants when each rally started and ended. According to our study protocol [44], only rallies with a duration ≥ 3 s were considered for subsequent analysis, because this guarantees that at least one complete ball exchange occurred within the rally, excluding initial serve. Then, EEG rally segments were defined as the segments of the EEG recordings starting 500 ms before the beginning of the rally and ending 500 ms after the end of the rally. Note that the duration of the rallies varied between conditions and subjects, as shown in Table 2.

Independent component analysis (ICA) was then used to separate brain data from physiological (eye blink, eye movement, cardiac, pulse, myogenic) and non-physiological artifacts (electrode jumps, interference from movements of the EEG cap cables, environmental interference) [47]. For each dyad and condition, ICA was applied to a unique EEG trial obtained by concatenating all EEG rally segments. Table 2 provides the total duration of the concatenated EEG trials. Each EEG trial was decomposed into 61 independent components (ICs) using the squared version of extended infomax, which proved to be effective for both super- and sub-Gaussian distributions [48]. The topography, time course, and spectral power of each IC were visually inspected, and the ICs related to artifacts were identified and disregarded. The denoised EEG trials, reconstructed by reprojecting the retained non-artifactual ICs onto the sensor space, were then filtered in the three frequency bands of interest: theta (4–8 Hz), alpha (8–12 Hz), and beta (12–30 Hz) and separated into EEG segments corresponding to the previously identified EEG rally segments.

Finally, the denoised EEG rally segments were prepared for the subsequent analysis: for each frequency band, they were Hilbert transformed to extract the instantaneous phase. To achieve a sufficient number of functional connectivity maps for statistical analysis, each Hilbert transformed denoised EEG rally segment was divided into non-overlapping windows of 500 ms, sufficiently long to guarantee a reliable functional connectivity analysis [49]. Given that different rallies had different durations, this procedure resulted in a different number of windows per rally.

#### 2.4.2. Functional Connectivity Estimation

For each window of each EEG rally segment, we pooled together the EEG data of the two members of the dyad, resulting in a 122 (electrodes) by 512 (samples) matrix of complex time series. This was carried out for each condition (cooperation and competition) and for each frequency band (theta, alpha and beta). Then, we estimated hyperbrain functional connectivity over this matrix, resulting in a 122 by 122 functional hyperconnectivity matrix. The corrected imaginary part of the phase locking value (ciPLV) [50] was used to estimate the phase synchronization between the two EEG signals, akin to PLV. However, ciPLV is highly computationally efficient and, unlike PLV [51], remarkably insensitive to zero lag synchronization, and therefore, it is highly robust to issues related to volume conduction effects [50]. We calculated ciPLV as follows:

(1) $${ciPLV}_{i,j}=\frac{\frac{1}{T}\left|I\left\{{xn}_{i,t}\cdot {xn}_{j,t}^{T}\right\}\right|}{\sqrt{1-{\left(\frac{1}{T}\left|R\left\{{xn}_{i,t}\cdot {xn}_{j,t}^{T}\right\}\right|\right)}^{2}}}$$

where ${xn}_{i,t}$ is the normalized value of the signal *x* related to the *i*th electrode at instant *t*; the complex signal *x*, as resulted from the Hilbert transformation, is normalized with respect to its complex magnitude; $I\left\{y\right\}$ and $R\left\{y\right\}$ stand for the imaginary and real parts of *y*, respectively.

**Figure 3:**
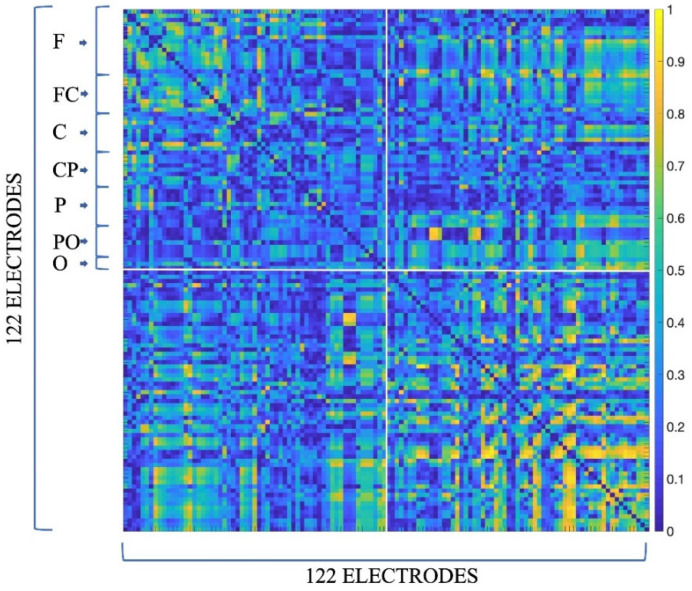
Example hyperbrain connectivity map of ciPLV values for the male dyad during cooperation, for the theta band. This is a square matrix of 122 electrodes per side (resulting from the sum of the 61 electrodes used for the EEG recordings of the 2 members of the dyad). Each group of 61 electrodes is ordered as follows: F—frontal electrodes; FC—fronto–central electrodes; C—central electrodes; CP—centro–parietal electrodes; P—parietal electrodes; PO—parietal–occipital electrodes; O—occipital electrodes.

As a result, we obtained hyperbrain functional connectivity maps, i.e., ciPLV matrices (see Figure 3) composed of 4 quadrants: the upper left quadrant is the within-brain functional connectivity map of member 1 of the dyad, the lower right quadrant is the within-brain functional connectivity map of member 2 of the dyad, and the upper right and lower left quadrants are the between-brain functional connectivity maps of the two members of the dyad. Note that while the within-brain functional connectivity maps are symmetrical, the two between-brain maps are not, with one between-brain map being the transposition of the other within the same hyperbrain functional connectivity map.

Thus, for each EEG rally segment, we obtained a total of N hyperbrain maps, where N is the number of time windows in the EEG rally segment. Each hyperbrain map was composed of 122 by 122 ciPLV values. This procedure was repeated for each dyad, frequency band, and interpersonal interaction condition.

Finally, we applied a threshold to the hyperbrain maps to retain only the strongest functional connections between electrodes. Given that different thresholds could lead to different study conclusions [52] and that no criterion has been determined so far to establish the optimal threshold, consistent with other studies [22,53], we opted for a fixed-cost threshold. We used a cost function of 0.2, retaining the 20% of connections with the highest ciPLV values. The other connections with lower ciPLV values (80% of the total connections) were set to 0.

#### 2.4.3. Graph Theoretical Measures

Graph theoretical concepts enable study of the topological features of the functional networks represented by the hyperbrain maps, where the ciPLV values represent the functional links (i.e., edges) between the electrodes (i.e., nodes). If a ciPLV value is zero, it means that the corresponding two electrodes are not functionally connected or, in the case of the thresholded maps, that those links are too weak to be considered. Graph theoretical measures were calculated to characterize the patterns of neural activations described in the functional connectivity maps and to differentiate between cooperation and competition, possibly highlighting differences across frequency bands within the same joint action condition.

To determine the role of nodes in the hyperbrain maps, we calculated strength and participation coefficient [54]. In graph theory, strength (*S*) is calculated for each node of a graph and estimates how strongly connected it is with other nodes in the graph. In other words, *S* detects zones of high concentration of edges in a graph. For the *i*th node of a graph, *S* is the sum of the weights of all edges connected to that node, where, in our case, the weights of the edges are quantified by the ciPLV values ($w_{ij}$) related to the *i*th node:

(2) $$S_{i}^{w}=\sum_{j=1}^{N}w_{ij}$$

*S* was determined for each node *i* of a thresholded hyperbrain map of ciPLV values (SH) and separately for the thresholded within- and between-brain maps (SWI and SBE, respectively). In accordance with Müller and colleagues (2013) [55], the SBE was calculated by subtracting the SWI values from the SH values.

Participation coefficient (*P*) quantifies the relation between the number of edges connecting a node outside its community and its total number of edges. Therefore, *P* is a measure of node edge distribution across the communities in a network. If a node’s edges are entirely restricted to its community, its *P* is 0, whereas when *P* is close or equal to 1, this node is strongly connected with nodes in other communities in the network. *P* for node *i* is defined as:

(3) $$P_{i}=1-\sum_{m=1}^{M}{\left(\frac{e_{im}}{e_{i}}\right)}^{2}$$

where *M* is the total number of modules (*m*) in the graph, $e_{im}$ is the number of edges between node *i* and all other nodes in module *m*, and $e_{i}$ is the total degree of node *i* in the network. Given that the participation coefficient can be evaluated only on symmetric matrices, it was calculated for only the thresholded hyperbrain maps.

To quantify the degree of integration, segregation, and efficiency of the functional brain networks [56,57,58,59] we also calculated local and global efficiency (LE, GE). To do so, we transformed the thresholded hyperbrain maps into binary adjacency hyperbrain matrices, where the retained connections were assigned a value of one, whereas the other connections were kept equal to zero. Given that LE and GE can be evaluated only on symmetric matrices, they were calculated for only the binary adjacency hyperbrain and within-brain matrices.

LE is a measure of segregation of a network, indicating that efficient information transfer occurs mainly in the immediate neighborhood of each node. LE also shows how fault-tolerant the network is, because the transfer of information across the network, being supported by multiple connections at the local level, can rely on alternative routes in case of a shortage of connections at the local level. LE is calculated as the harmonic mean of neighbor–neighbor distances:

(4) $$LE=\frac{1}{N}\sum_{j=1}^{N}{LEn}_{i}=\frac{1}{N}\sum_{j=1}^{N}\frac{\sum_{j,k\in G_{i}}\frac{1}{d_{jh}}}{N_{G_{i}}\left(N_{G_{i}}-1\right)}$$

where $N_{G_{i}}$ is the number of nodes in subgraph $G_{i}$, comprising all nodes’ neighbors of node *i* and excluding node *i* itself, and ${LEn}_{i}$ is the local efficiency of node *i* determined as the reciprocal of the shortest path length between neighbors *j* and *h*.

GE is a measure of integration in a network, indicating that efficient information transfer occurs also across distant nodes. GE is primarily influenced by short paths (i.e., connection paths between nodes). GE is defined as the average inverse shortest path length and is calculated as:

(5) $$GE=\frac{1}{N}\sum_{j=1}^{N}{NE}_{i}=\frac{1}{N\left(N-1\right)}\sum_{j=1,j\neq i}^{N}\frac{1}{d_{ij}}$$

where ${NE}_{i}$ is the nodal efficiency of the node *i* determined as the normalized sum of the reciprocal of the shortest path length from a given node to all other nodes in the network, and $d_{ij}$ is the shorthest path length between nodes *i* and *j*.

The list of graph theoretical measures calculated for each type of functional connectivity map (within, between and hyperbrain) is given in Table 3.

#### 2.4.4. Clustering Procedure

To identify specific functional patterns sustaining the two joint action conditions, we applied a clustering procedure to the within- and between-brain maps obtained for cooperation and competition in the three frequency bands. By doing so, we aimed at highlighting possible differences in the functional organization of the neural activations underpinning cooperation and competition.

For each dyad, interaction condition, frequency band, and time window of each EEG rally segment, the vectors of ciPLV values were extracted from the within- and between-brain maps. Given that the within-brain maps were symmetrical, we could extract a phase synchrony vector with 1 × 1830 size (within connectivity vector) from the upper triangular part of each map. Conversely, given that the between-brain maps were not symmetrical, a phase synchrony vector with 1 × 3721 size (between connectivity vector) was extracted from each map.

**Figure 4:**
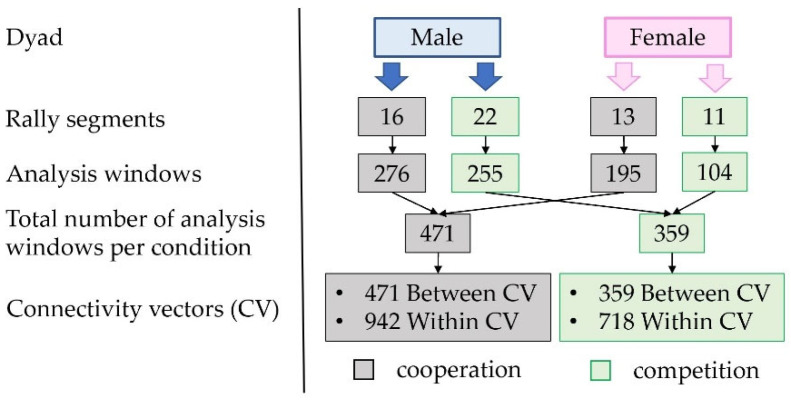
Procedure to obtain the connectivity vectors for each frequency band. The numbers listed refer to our proof-of-concept study and represent the connectivity vectors of between and within type, in cooperation and competition condition for each respective frequency band to which the clustering procedure was applied.

The within and between connectivity vectors were then grouped by condition and frequency band, resulting in 12 distinct groups of connectivity vectors. Figure 4 depicts how 4 groups of connectivity vectors were obtained for each frequency band, and how many connectivity vectors were available in our proof-of-concept study for the identification of the template maps for cooperation and competition and for the within- and between-brain maps.

The k-means algorithm [60,61] was applied to each group of connectivity vectors to identify characteristic functional maps of cooperation and competition in the three frequency bands. The k-means algorithm was preferred to other similar procedures because it is computationally efficient and can handle large datasets [60,62].

The optimal number of clusters (i.e., templates of functional connectivity vectors) was identified by repeating the k-means algorithm with *k* varying from 1 to 10 and estimating the Calinski–Harabasz criterion for each *k* value [63]; the optimal number of clusters corresponds to the *k* value for which the Calinski–Harabasz criterion, sometimes called the variance ratio criterion, provides the highest index value. Using this value of *k* ensures that clusters with large between-cluster variance and clusters with small within-cluster variance are well defined. The k-means clustering procedure [60,61] was applied to each group of connectivity vectors using the identified optimal *k*, and the functional connectivity templates were reconstructed for each group of connectivity vectors.

#### 2.4.5. Statistical Analysis

Statistical analysis was performed only on the graph metrics. No statistical analysis was performed on the results of the clustering procedure because no sufficient templates could be reconstructed from 2 dyads.

To detect significant topological differences between the functional connectivity maps obtained for cooperation and competition in the three frequency bands, the Wilcoxon rank sum test was separately applied to each graph metric evaluated for the within-brain, between-brain, and hyperbrain maps. The significance level was set at 0.05, and the statistical analysis was performed with a two-tailed approach. Separately for cooperation and competition and for each frequency band, the strength of each node was averaged across time windows and the EEG rally segments of the thresholded within- and between-brain maps. The 61 values of strength obtained per condition and frequency band were then compared.

Similarly, the participation coefficient of each node of the (122 × 122) thresholded hyperbrain maps was averaged across time windows and EEG rally segments separately for cooperation and competition and for each frequency band. The 122 values of participation coefficient obtained per condition and frequency band were then compared.

Finally, local and global efficiency of the binary adjacency hyperbrain and within-brain maps were averaged across windows separately for interaction condition and frequency band. The LE and GE values obtained per condition and frequency band were then compared between interaction conditions for each frequency band. Although the numbers of LE and GE values were quite small (38 values for cooperation and 24 values for competition), we applied the Wilcoxon rank sum test because it has been demonstrated to be robust for small samples [64].

To investigate potential functional correlations between strength and participation coefficient in the two experimental conditions, we performed a Pearson’s correlation analysis of the hyperbrain maps obtained for cooperation and competition in the three frequency bands, reporting the correlation coefficient r and the significance level (*p*-value) of the null hypothesis.

The Pearson’s correlation analysis was also applied to the results of the cluster analysis to investigate whether a tendency towards the predominance of one cluster over the other(s) occurred in the two interaction conditions.

## 3. Results

### 3.1. Graph Theoretical Measures

#### 3.1.1. Strength

The descriptive statistics of strength and the results of the two-tailed Wilcoxon rank sum test for cooperation and competition in each frequency band are reported in Table 4. The results highlight a significant difference between cooperation and competition for both within- and between-brain maps in the alpha and beta bands.

As expected, we observed that the average strength tended to decrease with the increasing frequency for both cooperation and competition regardless of the type of connectivity map. We can also see that, for cooperation, strength is reduced from the within- to the between-brain map in the theta band, whereas it remains almost unchanged for the alpha and beta bands. Conversely, for competition, we observed a reduction of the average strength from the within- to the between-brain maps for all frequency bands. It is also worth noting that strength in the alpha and beta bands during competition was higher in the case of cooperation in the within-brain maps, whereas it was lower for cooperation in the between-brain maps. The box plots for strength are reported in Figure S1.

**Figure 5:**
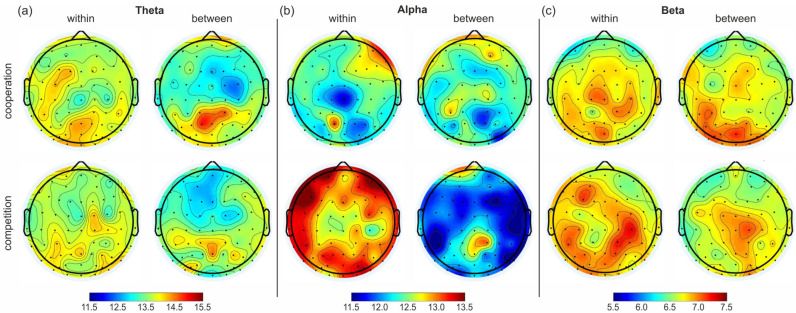
Topographical plots of average strength for the 61 nodes of the within- and between-brain matrices in the three frequency bands: (**a**) theta, (**b**) alpha, and (**c**) beta band. The left column of each subfigure shows the average strengths of nodes across all time windows and across all respective rallies in the weighted and thresholded within-brain matrices in the respective bands. The right column of each subfigure shows the results for the between-brain matrices in the respective bands. The upper row shows the results for the cooperation condition, whereas the bottom row shows the results for the competition condition, respectively.

Figure 5 shows the topographical plots for strength in the within and between matrices for cooperation and competition and the three frequency bands. The values of strength shown have been averaged for each node across the 4 members of the 2 dyads participating in our proof-of-concept study.

For the theta band (Figure 5a), no clear predominance of any node (i.e., electrode) in terms of links with other nodes was observed for the within-brain maps. Conversely, a significant increase of connections was observed in some prefrontal nodes and in the posterior area in the between-brain maps during cooperation, together with a small reduction in connections for the nodes in the central area. This effect was reduced in the between maps related to competition.

For the alpha band (Figure 5b) and the within-brain maps, a high level of connections was observed only in the right fronto–temporal electrodes during cooperation, whereas during competition, high levels of connections were observed for the frontal, temporal, parietal, and occipital areas. In the between-brain map for cooperation, the pattern of strength across nodes remained almost unchanged with respect to the within-brain map, whereas the between-brain map for competition showed a substantial decrease in strength for all nodes, with the exception of a few prefrontal electrodes.

In the beta band (Figure 5c), although all strength values were much lower than those in the other two frequency bands, we observed that in the within-brain maps, the nodes with higher numbers of connections were in the central and occipital areas for cooperation and in the temporal, parietal, and occipital areas for competition. In the between-brain map, during cooperation, we observed an increase of nodes with high numbers of connections in the occipital area with respect to the within-brain map, whereas the between-brain map during competition showed a reduction in strength with respect to both cooperation and the within-brain map for competition, with only some nodes in the central–parietal area having higher strength values.

#### 3.1.2. Participation Coefficient

The descriptive statistics of participation coefficient and the results of the two-tailed Wilcoxon rank sum test for cooperation and competition in each frequency band are reported in Table 5 and shown in Figure S2. The results highlight a significant difference between cooperation and competition in all frequency bands, with the participation coefficient always being higher during competition. We observed that the participation coefficient increased with increasing frequency during both joint action conditions, although more markedly during cooperation than competition. Finally, it is worth noting that the mean values of the participation coefficient were always greater than 0.45, indicating a relatively uniform link distribution with other nodes within the hyperbrain maps.

**Figure 6:**
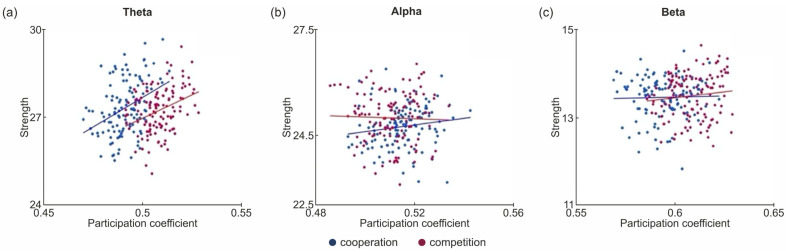
Scatterplots of Pearson’s correlation results of the associations between average participation coefficient and average strength of hyperbrain matrices, respectively, for each frequency band: (**a**) results for the theta band in cooperation vs. competition condition; (**b**) results for the alpha band in cooperation vs. competition condition; (**c**) results for the beta band in cooperation vs. competition condition. Lines indicate the direction of relationship between average participation coefficient and average strength in hyperbrain matrices.

To better explore how the roles of nodes varied between cooperation and competition across frequency bands, we calculated the Pearson correlation between participation coefficient and strength for the hyperbrain maps. Figure 6 shows the average participation coefficient (PC) and strength (S) values of individual hyperbrain matrices under the two experimental conditions and for the three frequency bands, on a PC-S plan.

The Pearson’s correlation test revealed that a significant positive correlation existed between participation coefficient and strength in the theta band for both conditions (see Table 6). This indicates that, for the theta band, an increase in participation coefficient is generally associated with an increase in strength in both cooperation and competition, although with higher values for both metrics during competition. This result may indicate that, for the same value of participation coefficient under the two experimental conditions (i.e., for the same type of functional organization of connections in the network), the quantity and robustness of connections during competition is higher than during cooperation. No significant correlation between participation coefficient and strength was found for the alpha or beta bands.

#### 3.1.3. Local and Global Efficiency

Table 7 shows the descriptive statistics and results of the Wilcoxon rank sum test for LE and GE for the binary adjacency hyperbrain and intra-brain maps. The same results are shown as box plots in Figure S3. The results of the Wilcoxon rank sum test show that a significant difference between cooperation and competition was found only for GE estimated on the within-brain maps in the alpha band (GE_coop_ = 0.693 ± 0.0141, GE_comp_ = 0.700 ± 0.0171; *p* = 0.007; z = 2.7017; effect size = 0.243). We can also observe that GE remained almost constant in both cooperation and competition in the within and hyperbrain maps and across frequency bands (GE ≈ 0.7). Conversely, LE, which was much lower than GE in the within-brain maps (mean LE ranging between 0.365 and 0.373), substantially increased in the hyperbrain maps, becoming greater than GE (mean LE ranging between 0.728 and 0.748), although this change was not significant. Overall, these results indicate that, at the level of individual functional networks, the flow of information is more efficient at a global than local level, whereas, at the level of hyperbrain networks, the flow of information becomes equally efficient at the global and local levels.

### 3.2. Results of the Clustering Procedure

In our proof-of-concept study, for cooperation, 942 and 471 connectivity vectors were available for the within- and between-brain maps, respectively, in each frequency band, whereas for competition, 718 and 359 connectivity vectors were available for the within- and between-brain maps, respectively, in each frequency band.

**Figure 7:**
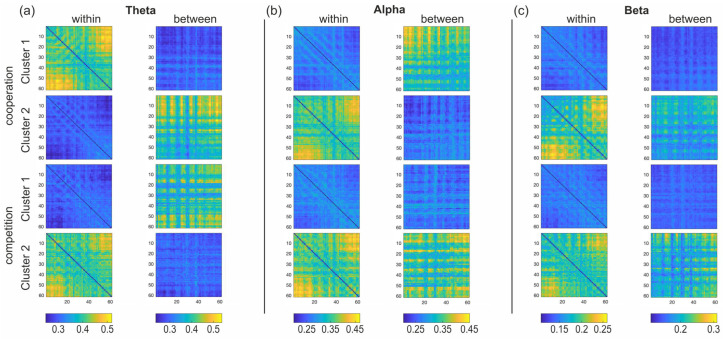
Plots of the canonical clusters obtained for the theta (panel **a**), alpha (panel **b**), and beta (panel **c**) bands. In each panel, the 4 upper clusters refer to cooperation and the lower 4 clusters to competition, and, for each interaction condition, the 2 clusters on the left-hand side refer to the within-brain matrices whereas the 2 clusters on the right-hand side refer to the between-brain matrices. In each cluster, the x and y axes refer to the electrodes, ordered as follows: frontal, fronto–central, central, centro–parietal, parietal, parietal–occipital, occipital electrodes from left to right in the x axis and from top to bottom in the y axis, respectively.

Based on the Calinski–Harabasz criterion, the optimal number of clusters for each group was *k* = 2. Therefore, two representative vectors of connectivity were extracted for each of the 12 groups of connectivity vectors. These representative vectors were then transformed into template connectivity maps (or canonical clusters) for the within- and between-brain maps for the three frequency bands considered. Figure 7 shows the two canonical clusters extracted for all conditions and frequency bands.

Due to the low number of subjects involved in our proof-of-concept study (two dyads, hence, four subjects), no statistical analysis could be performed to detect significant differences between the template connectivity maps characterizing cooperation and competition. However, we can observe that, for all frequency bands, distinct clusters were extracted for cooperation and competition, and these differences were more pronounced for the between-brain maps than for the within-brain maps. In particular, we observed that a higher number of connections between the parieto–occipital and frontal areas characterized the within-brain canonical maps in all frequency bands and conditions, whereas the between-brain canonical maps were characterized by a connectivity pattern that linked most brain areas of one member of the dyad with a reduced number of brain areas of the other member of the dyad.

To explore whether a predominant cluster could be identified, the Pearson’s correlation was computed between each template connectivity cluster extracted from each group and each connectivity vector in the same group. Table 8 reports for each member of each dyad the percentage of times that each cluster had a higher correlation than the other cluster with the connectivity vectors for each group. By doing so, we provide an idea of how representative one cluster can be of the functional patterns sustaining cooperation and competition in the different frequency bands. For instance, it occurred that one cluster was more representative than the other under both interaction conditions for one member of the dyad (theta band, cluster 1 for P1 in the female dyad; alpha band, cluster 1 for P2 in the female dyad; beta band, cluster 2 for P2 in the female dyad; beta band, cluster 1 for P2 in the male dyad), while, at times, the predominant cluster changed between cooperation and competition (theta band, cluster 2 to cluster 1 for P2 in the female dyad; theta band, cluster 2 to cluster 1 for P1 in the male dyad; beta band, cluster 2 to cluster 1 for P2 in the female dyad).

## 4. Discussion

The proposed pipeline was specifically conceived for the analysis of EEG-hyperscanning data recorded in a naturalistic environment and employing a turn-based motor task (i.e., table tennis) that is suitable not only for cooperative but also for competitive joint actions. However, this choice implies a revision of the traditional approaches used for both preprocessing and processing of EEG-hyperscanning data. First, the selection of a naturalistic type of interpersonal interaction implied the use of a motor task with the drawback of producing motion-related artefacts that may heavily affect the EEG recordings. Second, the unpredictability and variability of competitive (and turn-based) actions pose analytical challenges, rendering traditional analytical approaches less effective and imposing the identification of a novel analytical approach suitable to detect functional synchronization even in conditions of variable frequency of action.

To cope with the first problem, we designed a dedicated preprocessing pipeline which included both band-pass filtering and ICA decomposition. Employing a band-pass filter with a high-pass cutoff frequency set at 3 Hz, we can mitigate the effects of artifacts typically associated with body movements during intense and complex activities. In fact, although there are currently no guidelines or standardized approaches to manage motion-related artifacts in the EEG, previous studies have shown that motion artifacts mainly affect lower frequencies up to about 2 Hz for more intense and complex movements [65,66]. Therefore, the choice of a band-pass filter with a high-pass cutoff frequency of 3 Hz was determined by the intention to mitigate motion-related artifacts in the EEG recordings. In association with a low-pass cutoff frequency set at 40 Hz, our band-pass filter permits removal of most physiological and non-physiological interferences affecting the EEG without removing the neural frequencies relevant to the study of joint action, namely the theta, alpha, and beta bands. Indeed, the literature suggests that the application of both high-pass and low-pass filters enhances signal quality, especially for intentional movements [65,66]. Finally, the inclusion of an ICA-based preprocessing step permits decomposition of the EEG signals and identification of those signal components containing physiological and non-physiological artifacts that have frequency content included in the range between 3 and 40 Hz, making our denoising methodology more robust.

A novelty in our proposed processing step regards the use of an algorithm suitable for the analysis of turn-based competitive EEG-hyperscanning signals. In line with the EEG-hyperscanning literature on cooperative vs. competitive joint actions, we employed a zero-lag-insensitive, phase synchrony-based approach. Previous studies have usually employed the phase locking value (PLV) to estimate the phase synchronization between two EEG signals in the comparison between cooperation and competition [34,35,36]. Liu and colleagues (2021) [34] and Sinha and colleagues (2016) [35] did not obtain significant results, whereas Léné and colleagues (2021) [36] succeeded in differentiating cooperation from competition. However, it must be noted that Léné and colleagues did not use an ecological study paradigm, but a computer-based fast button-response task. Therefore, they did not demonstrate the applicability of PLV (or other functional connectivity algorithms) in the analysis of competition data acquired during the performance of an ecological motor task. Differently from these studies, we employed the corrected imaginary part of the phase locking value (ciPLV; [50]), a recently introduced measure of phase synchrony that offers several advantages in the study of neural coupling—particularly during competition—compared with PLV. These advantages include (1) high computational efficiency; in fact, the employment of ciPLV avoids some computationally expensive operations, achieving a 100-fold speedup over the PLV algorithm; (2) strong symmetry with coherence; ciPLV exhibits robust symmetry with coherence, one of the suggested approaches for studying competitive joint actions in the context of EEG hyperscanning [36]; (3) high robustness in the presence of volume conduction, along with the ability to ignore zero-lag connectivity while accurately estimating nonzero-lag connectivity [50].

From the EEG signals recorded during cooperation and competition, we obtained hyperbrain, within-, and between-brain maps representing the functional connectivity associated with the two interaction conditions. To highlight the main features of these maps, we employed four graph theoretical measures. Strength was used to identify the nodes with more connections with other nodes in the network, hence providing an indication of the relative functional importance of all nodes within a network. Participation coefficient is a measure of node edge distribution across the communities in a network; hence, it assesses the extent to which a node is functionally involved in various parts of the network rather than being strongly specialized in a specific community. High participation coefficients indicate that the various parts of a network are strongly interconnected, implying less specialization. This may reflect increased integration and information sharing among different areas of the neural network during social interactions. Conversely, low participation coefficients suggest that the different communities in a network are less interconnected, highlighting a greater functional specialization in different brain areas under specific social conditions. To further examine the interrelation between the number of connections per node and their distribution across the network during cooperation and competition, we calculated the correlation between strength and participation coefficient. Finally, the functional efficiency of the hyperbrain and within-brain networks was assessed in terms of network integration and segregation by calculating global and local efficiency (GE and LE), respectively. GE indicates how efficiently the transfer of information within a network occurs at a global level, whereas LE estimates the local efficiency of a functional network, namely, how efficiently a node communicates with its immediate neighbors.

To validate the proposed analytical pipeline, we performed a proof-of-concept study on two dyads playing table tennis, according to our recently published protocol [44]. We first observed a reduction in strength in the within- and between-brain networks from low frequencies (theta) to higher frequencies (beta) during both cooperation and competition. Indeed, recent studies in table tennis have highlighted a theta-dominance in anterior [67] and parieto–occipital areas [68], associated with increased visuomotor and cognitive demands. Our results, shown in the topographical plots of average strength for both within- and between-brain maps, are in agreement with these findings, revealing an increased theta engagement in the frontal and occipital brain areas. This result also aligns with the conclusions of Liu and colleagues [34], who emphasized the role of the theta band in interpersonal neural coupling during a motion-sensor racket task. Furthermore, Liu and colleagues [34] found increased alpha and beta activity in the occipital areas during cooperation, which decreased during competition. Our results also show an increased between-brain synchronization in the beta band within the occipital area that may be due to the shared attention and visual information processing between the cooperating subjects [69,70,71,72,73]. Moreover, the strength reduction observed for the alpha and beta bands in the between-brain maps during competition with respect to what was observed in the within-brain maps can be ascribed to a tendency of the members of the dyad to adopt different strategies during competition, when they focus more on their own performance [34,74].

The values of the participation coefficient obtained for the hyperbrain maps during competition indicate increased interconnections across various parts of the functional network compared with cooperation. This result suggests the occurrence of enhanced hyperbrain interactions during competition that may reflect improved information sharing, probably due to the need to cope with conflicts arising from heightened cognitive and motor demands. In contrast, the lower participation coefficient values obtained during cooperation may relate to the shared goal of establishing coordinated behavior, which aligns with the common private goal of keeping the ball in play for as long as possible, which requires lower cognitive and motor resources. The observation that higher values were found in the beta band could indicate that this frequency range is particularly relevant to the dynamics of competition. Indeed, the beta band is primarily associated with motor function; it is implicated in the planning, control, and execution of movements [75,76], particularly for complex and rhythmic motor tasks [77], fast-paced activities [78], and precision tasks [79]. Therefore, increased global interconnection in this band may be related to the more intricate, intense, and precise movements that characterize competition in comparison to cooperation.

The results of correlation between strength and participation coefficient indicated that enhanced node connectivity and the degree of interconnection among different network communities were positively correlated in the theta band for both cooperation and competition. This finding suggests that the presence of nodes with a high number of connections facilitates the occurrence of network-wide functional connections, emphasizing the role of the theta band in hyperbrain network dynamics. The finding that this correlation—although stronger during competition—existed in both interaction conditions aligns with previous results emphasizing that the theta band seems independent of the type of social interaction but is closely related to the nature of the task [67,68].

The calculation of global and local efficiency demonstrated that the hyperbrain maps were characterized by a more segregated than integrated functional efficiency across all frequency bands, in both cooperation and competition, with no significant differences between the two conditions. This result suggests that the integration and segregation of cortical areas in table tennis may be independent of the interaction condition. On the other hand, the within-brain networks exhibited greater integration than segregation, with alpha showing significantly higher integration during competition than cooperation. In agreement with our previous observations on strength and participation coefficients in cooperation and competition, the observed prevailing segregation of the hyperbrain networks might also support the notion that, during joint action, the interacting individuals were focused on coping with the planning, control, and execution of the complex, rhythmic and fast-paced movements required during competition, thus relying on more self-centered playing strategies. A similar result was found in our previous work on dyadic cooperative juggling [33], where the reduced global efficiency during interpersonal coordination was associated with increased task difficulty compared with individual juggling, suggesting that reduced global efficiency could be due to the increased difficulty associated with interactive juggling.

Another novel element in our analytical pipeline relates to the identification of functional connectivity clusters distinctive of cooperation and competition in the theta, alpha, and beta bands. By means of the k-means algorithm and Calinski–Harabasz criterion [60,61,62,63], we determined the optimal number of canonical clusters for each interaction condition and frequency band and extracted characteristic patterns of functional connectivity representative of the main neural activations sustaining cooperation and competition. Although the results obtained in our proof-of-concept study should be interpreted with caution because of the low number of analyzed subjects, distinct clusters for cooperation and competition were nonetheless extracted, providing an indication that this clustering approach can lead to interesting results in larger groups of subjects. For instance, the more intense functional connectivity observed between parieto–occipital and frontal areas in the within-brain canonical maps was in line with the results for strength and may suggest a predominant involvement of cognitive and sensorimotor areas to respond to the demands of the turn-based and competitive task. Conversely, the between-brain canonical maps highlighted connectivity patterns where most of the brain areas of one dyad member were linked with a reduced number of brain areas of the other dyad member. This pattern might indicate a leader–follower dynamic, potentially indicating how playing styles—such as offensive and defensive strategies, characterized by different behaviors and physiological responses [80,81]—may be reflected in distinctive functional connectivity patterns during joint action.

The low Pearson’s correlation values obtained when correlating the canonical clusters with each connectivity vector in the 12 groups underscore that a larger number of datasets should be analyzed. However, these correlation values still demonstrate that for each group of connectivity vectors, there is a general tendency towards the prevalence of one canonical cluster over the other. Interestingly, the predominant clusters generally differed between cooperation and competition within each group and frequency band. The predominance of one canonical cluster for a specific interaction condition and frequency band might be confirmed in a larger group of dyads, hence providing precious information on the prevalent functional connectivity patterns sustaining cooperation and competition.

## 5. Conclusions and Future Directions

The results of our proof-of-concept study were in line with the findings of other studies on competition, hence showing that the proposed analytical pipeline has the potential to effectively characterize and differentiate the neural dynamics sustaining different joint action conditions during the performance of naturalistic turn-based motor tasks. Based on the promising outcomes of the analysis of the EEG data recorded in the two dyads, we will employ the proposed analytical pipeline in a larger dataset of dyadic EEG recordings acquired during table tennis, once available.

One of the main challenges in the analysis of EEG–hyperbrain data collected during the execution of a motor joint action task relates to the removal of artifacts due to rapid body movements. In the proposed pipeline, we adopted a combination of band-pass filtering and ICA decomposition, which removed most large amplitude fluctuations. We are presently developing a novel analytical approach dedicated to the identification and removal of motion-related artifacts. The effectiveness of this approach will be tested in a large set of EEG-hyperscanning data and assessed in comparison with existing methods. Once established, this novel approach will be included in our proposed pipeline.

Possible future improvements of the proposed analytical pipeline also regard the identification and inclusion of other connectivity measures capable of elucidating the roles of players in a table tennis match. As suggested by our results on clustering, in joint action—especially during competition—a leader–follower interplay may be detected. The characterization of these roles could be beneficial for a deeper understanding of the neural basis of joint action. As well, the estimation of affective components through the analysis of psychophysiological signals such as heart rate variability or respiration rate [82], might lead to a more reliable comparison between cooperation and competition. These are already included in our table tennis study protocol [44] but were not collected during the proof-of-concept study, which focused on just EEG data analysis. Future dyadic acquisitions in table tennis will strictly follow our study protocol to provide a multimodal dataset on the multiple physiological activations during cooperative and competitive joint action, hence providing a valuable base for the testing and improvement of the proposed analytical pipeline.

## Figures and Tables

**Table 1:** Specifications of the dyadic EEG dataset. From left to right: number of dyadic EEG datasets, gender of dyads, age of subjects for each dyad, sampling frequency of EEG recording for each dyad, recording’s condition, total duration of the EEG recording for each subject and condition.

N. Dyadic EEG Datasets	Gender	Age (year)	EEG Sampling Frequency (Hz)	Recording’s Condition	Total Recording Duration (m)
2	male	18	1024	cooperative	8.14
		31		competitive	16.27
	female	19		cooperative	8.27
		21		competitive	16.53

**Table 2:** Descriptive statistics of the rallies played in cooperative and competitive mode. From left to right: condition, dyad gender, total number of rallies, mean ± STD of rally duration, range of rally duration, median of rally duration, 5th and 95th percentiles of rally duration, total duration of the concatenated EEG trials.

Condition	Dyad Gender	No. of Rallies	Mean ± STD (s)	Range (s)	Median (s)	5th and 95th Percentiles (s)	Total Duration of Concatenated EEG Trials (s)
COOP	male	16	7.80 ± 4.20	(3.30 ÷ 18.70)	6.50	3.45; 17.11	157.00
	female	13	6.70 ± 3.40	(3.50 ÷ 12.60)	5.40	3.52; 12.48	113.40
COMP	male	22	5.00 ± 1.40	(3.20 ÷ 8.20)	4.85	3.32; 7.54	153.90
	female	11	3.90 ± 1.50	(3.00 ÷ 7.40)	3.40	3.01; 7.35	65.30

**Table 3:** List of graph theoretical measures calculated for within, between, and hyperbrain maps. Participation coefficient was computed for weighted and thresholded hyperbrain matrices; strength was computed for weighted and thresholded within, between, and hyperbrain matrices; LE and GE were computed for only binary intra-brain matrices.

Graph Theoretical Measure	Within Matrices	Between Matrices	Hyperbrain Matrices
Participation coefficient			✓
Strength	✓	✓	✓
Global efficiency (GE)	✓		✓
Local efficiency (LE)	✓		✓

**Table 4:** Descriptive statistics and results of the Wilcoxon rank sum test on strength calculated on the within- and between-brain maps. From left to right: type of connectivity map, type of joint action condition, and for each frequency band: mean ± STD, median (5th and 95th percentile), *p*-value of the two-tailed Wilcoxon rank sum test computed at a significance level of 0.05.

		Theta			Alpha			Beta		
Connectivity Map	Condition	Mean ± STD (s)	Median (Percentiles) (s)	*p*	Mean ± STD (s)	Median (Percentiles) (s)	*p*	Mean ± STD (s)	Median (Percentiles) (s)	*p*
Within	COOP	13.82 ± 0.28	13.80 (13.44; 14.24)	0.34	12.37 ± 0.26	12.35 (11.95; 12.85)	<0.001	6.72 ± 0.17	6.72 (6.43; 7.00)	<0.01
	COMP	13.77 ± 0.26	13.75 (13.32; 14.17)		13.04 ± 0.29	13.07 (12.57; 13.56)		6.83 ± 0.17	6.83 (6.55; 7.12)	
Between	COOP	13.51 ± 0.63	13.50 (12.51; 14.42)	0.46	12.37 ± 0.32	12.36 (11.80; 12.88)	<0.001	6.75 ± 0.30	6.78 (6.14; 7.15)	<0.01
	COMP	13.46 ± 0.53	13.49 (12.60; 14.39)		11.96 ± 0.47	11.97 (11.26; 12.74)		6.68 ± 0.21	6.66 (6.33; 7.04)	

**Table 5:** Descriptive statistics and results of the Wilcoxon rank sum test on participation coefficients. From left to right: type of connectivity map, type of joint action condition; and for each frequency band: median of strength across nodes, 5th and 95th percentiles of strength, *p*-value of the two tailed Wilcoxon rank sum test computed at a significance level of 0.05.

		Theta			Alpha			Beta		
Connectivity Map	Condition	Mean ± STD (s)	Median (Percentiles) (s)	*p*	Mean ± STD (s)	Median (Percentiles) (s)	*p*	Mean ± STD (s)	Median (Percentiles) (s)	*p*
Hyperbrain	COOP	0.50 ± 0.01	0.50 (0.48; 0.51)	<0.001	0.51 ± 0.01	0.51 (0.49; 0.52)	<0.001	0.57 ± 0.01	0.57 (0.55; 0.60)	<0.001
	COMP	0.55 ± 0.01	0.55 (0.53; 0.57)		0.55 ± 0.01	0.55 (0.54; 0.58)		0.60 ± 0.01	0.59 (0.57; 0.61)	

**Table 6:** Pearson correlation results of relation between participation coefficient and strength. From left to right: condition of interaction, Pearson value (R), *p*-value (*p*) with a significance level of 0.05, frequency bands.

Condition		Theta	Alpha	Beta
COOP	R	0.37	0.15	0.03
	*p*	<0.001	0.09	0.80
COMP	R	0.35	−0.03	0.11
	*p*	<0.001	0.73	0.22

**Table 7:** Descriptive statistics and results of Wilcoxon rank sum test for LE and GE. From left to right: type of connectivity map, graph metric, type of joint action condition; and for each frequency band: mean ± STD, median, 5th and 95th percentiles, *p*-value of the two-tailed Wilcoxon rank sum test computed at a significance level of 0.05.

			Theta		Alpha		Beta	
Connectivity Map	Metric	Condition	Mean ± STD (s)	*p*	Mean ± STD (s)	*p*	Mean ± STD (s)	*p*
			Median (Percentiles) (s)		Median (Percentiles) (s)		Median (Percentiles) (s)	
Within	LE	COOP	0.372 ± 0.010	0.308	0.372 ± 0.007	0.594	0.365 ± 0.009	0.691
			0.373 (0.354; 0.383)		0.372 (0.363; 0.385)		0.366 (0.351; 0.380)	
		COMP	0.371 ± 0.009		0.373 ± 0.009		0.365 ± 0.009	
			0.372 (0.355; 0.385)		0.372 (0.360; 0.388)		0.366 (0.349; 0.378)	
	GE	COOP	0.689 ± 0.021	0.647	0.693 ± 0.014	0.007 **	0.698 ± 0.014	0.257
			0. 694 (0.645; 0.715)		0. 693 (0.671; 0.721)		0. 699 (0.677; 0.723)	
		COMP	0.693 ± 0.019		0.700 ± 0.017		0.701 ± 0.016	
			0. 693 (0.660; 0.721)		0. 699 (0.672; 0.731)		0. 700 (0.672; 0.727)	
	LE-GE	COOP		<0.001		<0.001		<0.001
		COMP		<0.001		<0.001		<0.001
Hyperbrain	LE	COOP	0.748 ± 0.006	0.672	0.744 ± 0.005	0.662	0.729 ± 0.005	0.544
			0. 749 (0.739; 0.757)		0. 744 (0.735; 0.751)		0. 728 (0.721; 0.738)	
		COMP	0.748 ± 0.007		0.745 ± 0.005		0.728 ± 0.004	
			0. 747 (0.739; 0.759)		0. 745 (0.734; 0.752)		0. 728 (0.723; 0.736)	
	GE	COOP	0.698 ± 0.001	0.563	0.699 ± 0.001	0.622	0.700 ± 0.001	0.050
			0. 698 (0.696; 0.700)		0. 699 (0.698; 0.700)		0. 700 (0.700; 0.700)	
		COMP	0.698 ± 0.001		0.699 ± 0.001		0.700 ± 0.001	
			0. 698 (0.696; 0.700)		0. 699 (0.698; 0.700)		0. 700 (0.700; 0.700)	
	LE-GE	COOP		<0.001		<0.001		<0.001
		COMP		<0.001		<0.001		<0.001

**Table 8:** Pearson’s correlation between connectivity vectors and representative clusters. In the first row, the two experimental conditions. From left to right: frequency band, dyad gender, subject in each dyad, percentage of connectivity vectors represented by cluster 1 and cluster 2 in each group of connectivity vectors (per experimental condition, frequency band, and subject in the dyads).

	Dyad Gender	Subject	Cooperation		Competition	
			Cluster 1 (%)	Cluster 2 (%)	Cluster 1 (%)	Cluster 2 (%)
Theta	female	P1	60	40	61	39
		P2	45	55	57	43
	male	P1	41	59	54	46
		P2	50	50	57	43
Alpha	female	P1	45	55	50	50
		P2	57	43	55	45
	male	P1	62	38	44	56
		P2	45	55	51	49
Beta	female	P1	44	56	46	54
		P2	43	57	53	47
	male	P1	50	50	57	43
		P2	56	44	54	46

## Data Availability

The EEG datasets used in this study are available online at the Figshare repository via the following links: https://figshare.com/s/93fe79bf39683c61d4b7; https://figshare.com/s/a8d3475b16f590e7f945, accessed on 20 December 2023.

## Supplementary Materials

The following are available online at https://figshare.com/s/89bc32b86d46162cd6b3, accessed on 3 January 2024, Figure S1: Box plots of global strengths for the within- and between-brain matrices, Figure S2: Box plots of global participation coefficient for the hyperbrain matrices, Figure S3: Box plots of local efficiency (LE) and global efficiency (GE) for the within-brain and hyperbrain matrices.
